# Visual Attention Model Based on Statistical Properties of Neuron Responses

**DOI:** 10.1038/srep08873

**Published:** 2015-03-09

**Authors:** Haibin Duan, Xiaohua Wang

**Affiliations:** 1 State Key Laboratory of Virtual Reality Technology and Systems, Beihang University, Beijing 100191, P. R. China; 2 Science and Technology on Aircraft Control Laboratory, School of Automation Science and Electronic Engineering, Beihang University, Beijing 100191, P. R. China

## Abstract

Visual attention is a mechanism of the visual system that can select relevant objects from a specific scene. Interactions among neurons in multiple cortical areas are considered to be involved in attentional allocation. However, the characteristics of the encoded features and neuron responses in those attention related cortices are indefinite. Therefore, further investigations carried out in this study aim at demonstrating that unusual regions arousing more attention generally cause particular neuron responses. We suppose that visual saliency is obtained on the basis of neuron responses to contexts in natural scenes. A bottom-up visual attention model is proposed based on the self-information of neuron responses to test and verify the hypothesis. Four different color spaces are adopted and a novel entropy-based combination scheme is designed to make full use of color information. Valuable regions are highlighted while redundant backgrounds are suppressed in the saliency maps obtained by the proposed model. Comparative results reveal that the proposed model outperforms several state-of-the-art models. This study provides insights into the neuron responses based saliency detection and may underlie the neural mechanism of early visual cortices for bottom-up visual attention.

The visual system is equipped with outstanding capabilities of comprehending complex scenes rapidly and obtaining the key points that capture interest immediately^1^. Since the neuronal hardware is insufficient for such challenging tasks, an accurate encoding strategy with the minimal consumption of biological resources^2^,^3^ is evolved in the visual system. Visual attention is an important signal processing mechanism that can selectively increase the responses of cortical neurons that represent relevant information of potentially important objects^4^,^5^. The neural spiking activity and joint properties of neurons in early areas^6^ can be modulated by visual attention and thus only a crucial subset of input information is collected by intermediate and higher visual processes.

The visual attention mechanism has attracted many researchers in areas of psychology^7^,^8^,^9^, neurophysiology^10^,^11^, computational neuroscience^12^,^13^, and computer vision science^14^,^15^,^16^,^17^. Numerous areas in the cortex are related to attentional allocation, according to psychology and neurophysiology researches. Experiments in humans found that activities of the lateral geniculate nucleus (LGN) can be modulated by both spatial attention and saccadic eye movements^19^. The superior colliculus (SC) is the first structure subjected to strong attention effects^4^. Studies indicated the appearance of bottom-up saliency maps in the primary visual cortex (V1)^10^,^20^,^21^,^22^,^23^,^24^. However, there is also another study that could not find such attention modulation effects in human V1 area^25^. A possible explanation for this contrary result is that the task was not demanding enough to generate strong modulation of neuron responses^26^. Additionally, bottom-up visual saliency can be guided by the top-down information from deeper layers^27^,^28^,^29^,^30^,^31^ such as the lateral intraparietal (LIP) region of the cortex^29^,^30^, the frontal eye fields (FEF)^31^,^32^, and the pulvinar^33^,^34^. The areas LIP, FEF, pulvinar, the inferotemporal cortex (IT), and the feature-selective visual areas V2 and V4 all drive bottom-up attention signals and receive top-down attention biasing signals^20^,^29^,^30^,^31^,^32^,^33^,^34^ simultaneously. The medial temporal (MT), the medial superior temporal (MST), and the prefrontal cortices are modulated by both spatial and feature-based attention^35^,^36^.

Literatures analyzed above suggest that area V1 is important for the conformation of bottom-up saliency while other areas receive signals from both area V1 and deeper layers simultaneously. Neurons in area V1 can encode principal and independent components extracted from images^37^ efficiently. The response magnitude of neurons in V1 is higher when the stimulus is distinct from its surroundings^23^,^37^. Objects that stand out from their surroundings are generally considered to be salient and attention attracting^17^,^20^,^24^. However, the specific relationship between V1 neuron responses and visual attention is still not clear^38^,^39^. Therefore, we focus on the effect of area V1 upon bottom-up visual attention in free-viewing scenes.

We suppose that unique neuron responses imply salient image regions and more extraordinary neuron responses correspond to more salient regions, based on the analysis of statistical properties of neuron responses. Consequently, neuron responses to each location in scenes can be formed to predict the corresponding value of saliency. An integral visual attention model is proposed based on the statistical properties of neuron responses in area V1 to test the hypothesis. Firstly, V1 neuron responses to each pixel are calculated with the stimuli and a set of well-trained Gabor-like receptive fields of V1 neurons. Then a statistical distribution model of neuron responses is built to compute the possibility that a specific neuron response emerges. Afterwards, the saliency value of each pixel is obtained according to the self-information of the corresponding neuron responses.

Self-information is adopted to measure the saliency value according to emergence possibilities of neuron responses. This strategy was firstly used in the model of Attention based on Information Maximization^40^ (AIM). In AIM a saliency map was thought of as the average Shannon self-information of cells across a cortical column corresponding to content appearing at each spatial location. Here, we take a distinct approach. The statistical properties of neuron responses are considered and obtained based on interactions among the excitatory and inhibitory neurons in area V1. Color information in images should be completely used due to the fact that salient regions often have distinctive colors compared to the background. Therefore, four different color spaces including RGB, CIELAB, HSI, and YIQ are used in the proposed model to provide more integrated information. Saliency sub-maps of images in gray-scale and each separate channel of the color spaces are computed independently. Saliency sub-maps with lower entropies are selected and combined into the final saliency map using a novel entropy-based strategy.

The proposed visual attention model is tested on traffic images to highlight vehicles out of complex backgrounds. Comparative experiments are carried out against both excellent salient object detection models and eye fixation prediction models, including Chang's model (SVO)^41^, Goferman's model^42^, Jiang's model (CBsal)^43^, AIM^40^, the spectral residual (SR) approach^44^, and ITTI's model^14^. Furthermore, all the models are evaluated quantitatively using four popular visual attention datasets to demonstrate the superiority and stability of the proposed model.

## Results

**Figure 1:**
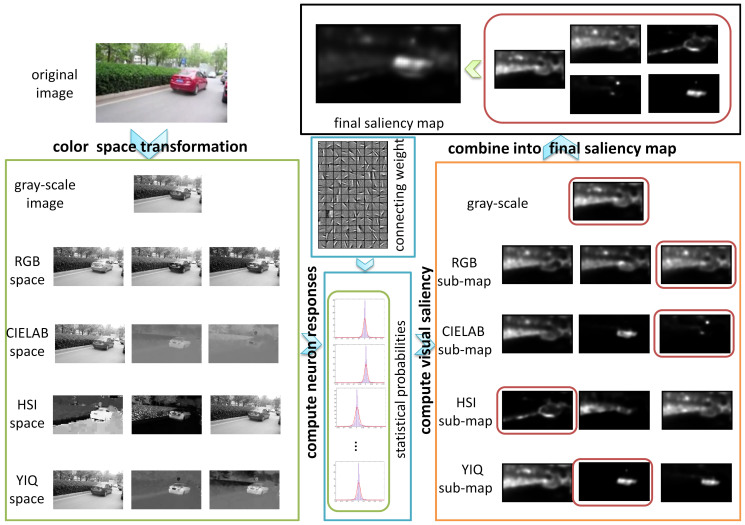
Framework of the proposed visual attention model. Each rectangle depicts an operation involved in the overall computational framework. ***Color space transformation***: Original image saved in the RGB color space is transformed into gray-scale image. It is also transformed into the CIELAB, HSI, and YIQ color spaces. Channels of all the four color spaces are separated. ***Compute neuron responses***: Neurons responses in area V1 are extracted from local patches of gray-scale image and all the twelve separate channels of the four color spaces (see Methods). Afterwards, statistical probabilities of the neuron responses are estimated based on the histogram of the neuron responses. ***Compute visual saliency***: Saliency sub-maps are obtained by adding the self-information in each dimension of the neuron responses up. Saliency sub-maps are computed for gray-scale image and all the twelve separate channels. ***Combine into final saliency map***: The entropy of each saliency sub-map is computed. Afterwards the sub-map with the lowest entropy is selected from each color space (the sub-maps in red rectangles). Finally, the selected sub-maps of all the four color spaces together with the sub-map of the gray-scale image are combined into the final saliency map. Reciprocals of their corresponding entropies are taken as combining weights for sub-maps. The original image is taken by X.H. Wang with a digital camera Canon IXUS 125HS.

As Figure 1 suggests, appearances in different color spaces reveal distinct information of an image. Four color spaces are used in the proposed model to take advantages of color information. The saliency sub-map in each channel is computed on the foundation of corresponding neuron responses. The final saliency map is obtained via a novel entropy-based combination operation. The red car in Figure 1 is highlighted while the plants and road in the background are suppressed in the final saliency map, revealing the effect of visual attention.

### Neuron dynamic spiking and statistical properties of neuron responses

Neurophysiological evidence certificates that neurons in early visual areas extract primary features and transmit them to high-level neurons of the visual cortex^37^. Receptive Fields (RFs) of neurons in the early visual cortex could be typically simulated by localized and oriented feature detectors, such as Gabor filter^45^ and Derivative of Gaussian (DoG) filter^46^. Sparse coding has ability to represent the properties of V1 neuron responses. For instance, SAILnet^47^ and E-I Net^48^ are coding strategies that are sufficient to account for the properties of V1 neurons. The outputs of the coding strategies are with sparseness and a high degree of statistical independence. There are two types of neurons in area V1: excitatory neurons and the inhibitory ones^48^. Inhibition and suppression functions between these two kinds of neurons are important mechanisms in the cortex^49^. Spiking rates of excitatory neurons can form a sparse representation of the input image patch. The learning framework of connecting matrixes (i.e. RFs) for inhibition and excitatory neurons are given in the Methods section. The neuron responses for a specific stimulus are determined after the connecting weights among the neurons have been learned.

**Figure 2:**
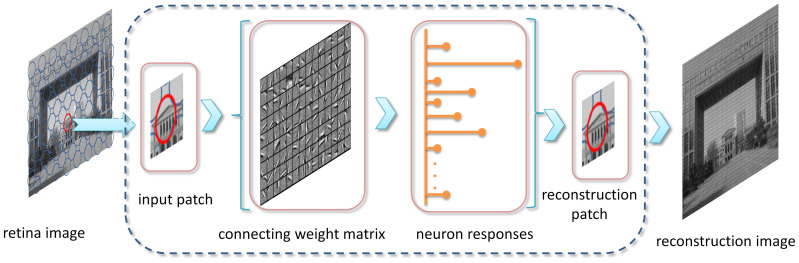
Image reconstruction with dynamic spiking activities of neurons. Patches are sampled from the retina image and imported to the learned encoding neural network. A patch can be reconstructed with a production of the excitatory neuron responses and the learned connection weights (see Method). The retrieve of the whole image can be obtained with each patch reinstated. The original image is taken by X.H. Wang with a digital camera Canon IXUS 125HS.

The number of spikes generated by each excitatory neuron represents a feature of the input stimuli with the well-learned connecting weights. The dynamic neuron spiking activities can be decoded to recover the input stimuli approximately. An image reconstruction experiment is conducted to verify the performance of the dynamic spiking layer and the results are presented in Figure 2.

**Figure 3:**
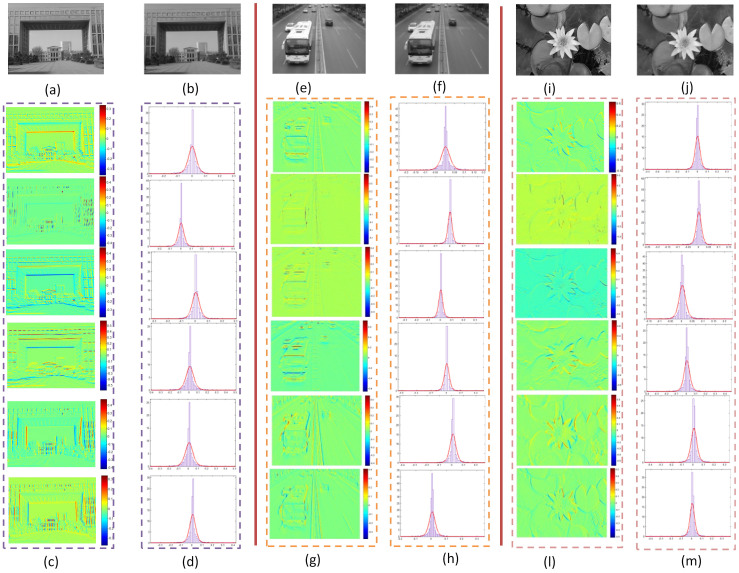
Statistical properties of neuron responses to retina images. (a), (e), (i) are original retina images; (b), (f), (g) are reconstructions of the original retina images; (c), (g), (l) from top to down are the 17th, 33th, 49th, 65th, 81st, and 97th dimensions of neuron responses projected to corresponding pixels of the original retina images, respectively; (d), (h), (m) from top to down are statistical histograms of the 17th, 33th, 49th, 65th, 81st, and 97th dimensions of neuron responses with their outlines fitted by red curves. The horizon rows represent values of the neuron responses and the vertical ones are numbers of neurons with corresponding responses. The original images given in (a), (e), (i) are taken by X.H. Wang with a digital camera Canon IXUS 125HS.

As shown in Figure 2, patches sampled from the input image are adopted as stimuli to neurons with the trained connection weights. The original image can be reconstructed with a production of the neuron responses and the learned connection weights. The simulation verifies the preciseness of neuron responses. Sampled neuron responses to three different scenes are shown in Figure 3, to investigate the statistical properties of the neuron responses.

All the three scenes are reconstructed accurately, which demonstrates the correctness of neuron responses. As shown in Figure 3 (d), (h), and (m), responses of most neurons are around zero and the numbers of larger responses decline quickly. Patches with more details such as edges of the building, car, and flower generally cause higher responses, while patches of smooth background such as the ground, sky, and road arouse lower ones. For example, almost all the neuron responses to the road in Figure 3 (g) are zero. By analyzing one dimension of the responses, it can be found that responses to similar stimuli are similar, while those to different stimuli are distinct. Furthermore, different dimensions of the neuron responses reflect distinct information. For instance, the 65th dimension of responses for all the three scenes emphasizes horizontal information while the 97th dimension emphasizes vertical information. Therefore, the properties of neuron responses indicated by our investigations are consistent with the real neuron responses of area V1^18^,^37^,^40^,^48^. The probability distributions of responses with high peaks near zero and long tails can be approximately described with the Generalized Gaussian Distribution (GGD) in addition to statistical histograms.

### Saliency extraction

The visual attention mechanism is suggested to be implemented by modifying the connectivity in specific cortex areas or by conducting particular temporal patterns of activities^50^,^51^. However, there are many factors that influence the conformation of visual attention. Low level features extracted from the input image are always employed to measure saliency^52^. The proposed bottom-up model adopts the information of features extracted by the excitatory neurons. Objects that differ from the rest of the scene maximally are regularly focuses of attention in a free view. Thus, we make a hypothesis that responses of the excitatory neurons are rare when the stimuli patch is a salient object. In other words, the saliency value of a patch is supposed to be high when the corresponding neuron responses appear with low probabilities. According to this hypothesis, the computation of a saliency map can be built on the basis of statistical probabilities of the corresponding neuron responses. Statistical probabilities can be computed with statistical histograms or with fitted GGD curves. A saliency map is computed as a linear combination of the salient values calculated by the response of each neuron as responses of all the excitatory neurons are approximately independent. Self-information can measure the likelihood that a random variable appears. Therefore, the saliency value of an input image can be calculated with the self-information of the corresponding neuron responses.

Color information is ignored since the RFs are trained with whiten images in gray scale. Twelve separate channels of four color spaces (i.e., RGB, CIELAB, HSI, and YIQ) are employed to provide different information for color images. Visual attention aims at extracting regions with high preciseness and suppress background and disturbance automatically. A valuable saliency map is supposed to be with low entropy as the contrast between the attentional focus and the background should be maximized. Thus, saliency sub-maps of different color channels can be selected and combined according to their entropies.

**Figure 4:**
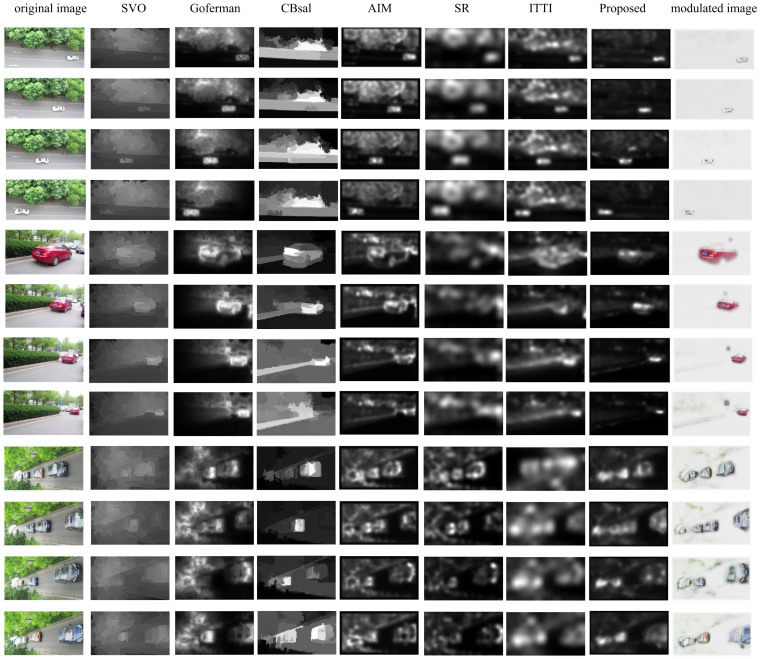
Experimental results of the seven models tested with traffic images. The first column shows the original images; the second to eighth columns present the saliency maps obtained by SVO, Goferman's model, CBsal, AIM, SR, ITTI's model, and the proposed model, respectively. Images modulated by the corresponding saliency maps obtained by the proposed method are given in the last column. The original images are all taken by X.H. Wang with a digital camera Canon IXUS 125HS.

Several traffic images are employed to test the performance of the proposed method together with SVO^41^, Goferman's model^42^, CBsal^43^, AIM^46^, SR^44^, and ITTI's model^14^. Experimental results of three instances are presented in Figure 4. The experiments are all conducted in Matlab R2012b running on a 3.2 GHz Intel Core i5 PC equipped with a 4 Gb RAM. Comparative results illustrate the differences among the seven different models. SVO and CBsal obtain object-level saliency as they take objectness information into consideration. Other methods attain pixel-level saliency. For the first case CBsal could not extract the object accurately since the white car is relatively small. For the second case with a red car whose size is larger, almost all the methods can make good performances. However, the performance of CBsal gets worse when the object becomes smaller. Additionally, for the last instance, the plants are highlighted in the saliency maps obtained by SVO, Goferman's model, AIM, SR, and ITTI's model. Moreover, Goferman's model, AIM, SR, and ITTI's model are short of inhibiting the redundant background. The attentional focuses in the saliency maps obtained by these four methods are not very compact. On the contrary, the proposed model can make the attentional focuses centralized at the regions of targets. The cars are intently highlighted by the proposed method as shown in Figure 4. But in the saliency maps obtained by other methods the plants are also turned out to be highlighted. Therefore, the proposed model reveals superiority in evidence. Images modulated by corresponding saliency maps that are obtained by the proposed model are also shown in Figure 4. In modulated images the most salient regions are reserved while the backgrounds are abandoned.

Furthermore, all the seven visual attention models are quantitatively evaluate using four public image saliency datasets: the dataset derived by Achanta R. et al.^53^, the DUT-OMRON dataset^54^, the Extended Complex Scene Saliency Dataset (ECSSD)^55^, and the THUS 10000 dataset^56^. The first dataset contains 1000 images together with fixation maps for all the images. The second one has 5168 images, the third one has 1000 images, and the last one contains 10000 images. The later three datasets all have manually segmented salient object masks for all the corresponding images. Human fixations in the fixation maps of the first dataset and salient objects in the manually segmented masks of the later three datasets are considered as the positive set and other pixels are in the negative set. Afterwards, several scores to quantitatively evaluate the visual attention models can be computed.

**Figure 5:**
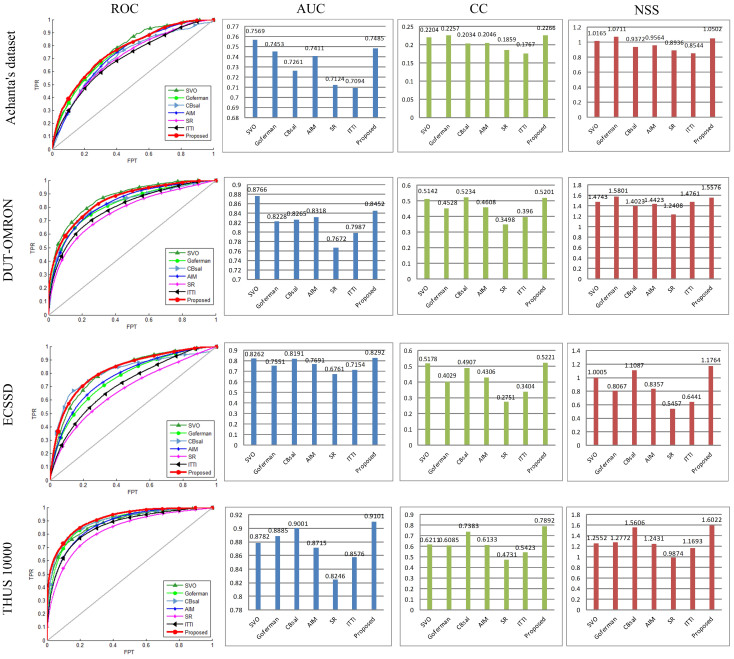
ROC, AUC, CC, and NSS scores of the seven models on four datasets. It should be mentioned that the number of tested images is set as 300 for each dataset, i.e. 300 images are randomly selected from each dataset to calculate the four scores for each model. Therefore, the scores might be somewhat different from those caculated with the whole datasets. The gray diagonal line in each ROC figure is the baseline, which presents the ROC curve of a random classification.

The saliency map of each image can be considered as a binary classifier which divides pixels in the original image into salient pixels versus not salient ones. A Receiver Operating Characteristic (ROC) curve^5^ can be generated for each visual attention model by choosing several different thresholds for classification and plotting True Positive Rate (TPR) vs. False Positive Rate (FPR). ROC curves for all the models are shown in Figure 5. Afterwards, Areas Under the ROC Curves (AUC) can be calculated. The perfect saliency prediction corresponds to an AUC score of 1 and a higher AUC score represents a better visual attention model. AUC score has an admirable property of transformation invariance in that this measure does not change when applying any monotonically increasing function to the saliency values^5^. Moreover, the Linear Correlation Coefficient (CC)^5^ and Normalized Scanpath Saliency (NSS)^5^ scores are also calculated and the results are given in Figure 5. The CC score measures the strength of a linear relationship between two variables. A CC score closed to +1/−1 indicates a perfect linear relationship between the ground-truth mask and the saliency map. NSS is defined as the average of the values at human eye positions in a normalized saliency map with zero mean and unit standard deviation. A NSS score lager than 1 indicates that the saliency values at human fixated pixels are significantly higher than other locations.

As shown in Figure 5, the AUC, CC and NSS scores obtained by the proposed model are all larger than those gained by AIM, SR, and ITTI's model. These results reveal the significant superiority of the proposed model on all the four datasets. The scores obtained by ITTI's model are mostly greater than those obtained by SR, but it is lower than those obtained by AIM. SVO acquires best experimental results on Achanta's dataset and the DUT-OMRON dataset. The proposed model performs significantly better than almost all the compared algorithms on the ECSSD dataset and the THUS 10000 dataset. The four scores calculated on the Achanta's dataset are generally lower than those calculated on other three datasets. The reason could be that the ground-truth images in this dataset are given as fixation maps, while those in the other three datasets are given as manually segmented salient object masks. Furthermore, results presented in Figure 5 show that the tendencies of ROC and AUC are more consistent. Since the CC and NSS scores are sensitive to center-preference, ROC and AUC are mainly considered to draw conclusions. Thus, SVO, the proposed method, and CBsal rank as the top three in order according to the average AUC score.

## Discussion

In this study we proposed a novel visual attention model based on statistical properties of neuron responses to test the hypothesis that area V1 is a potential structure for bottom-up visual attention. The statistical regularity of neuron responses is investigated. We found that the responses of different neurons are independent and the responses for similar stimuli are approximate while those for different stimuli are distinct. The neuron responses in these regions are suggested to be rare as interesting regions are appear special and distinct from the background. Self-information is adopted to calculate the saliency map. Additionally four different color spaces (i.e., RGB, CIELAB, HSI, and YIQ) are utilized. The saliency sub-map with the lowest entropy is selected from the three channels in each color space. The four selected saliency sub-maps of the color spaces together with the saliency map of the gray-scale image are combined. The corresponding reciprocals of entropies are adopted as weights for combination.

The distinctive aspects of the proposed model can be generalized as follows. Firstly, our model considers about both current scene and an ensemble of natural scenes, which is different from the models that only use information of current scene. Neuron responses are computed based on current image together with an ensemble of natural scenes in our model as RFs of neurons are learned from a set of patches sampled from natural images. Therefore, the computation of neuron responses is in accordance with human experience during evolution and development. Secondly, several different color channels are utilized in the proposed model differing from most models that merely conduct feature extraction in one color space. We used four color spaces to exploit color information as color perception in the visual system is uncertain. Thirdly, the utilization of entropy and the combination method are also unique. The saliency sub-maps with the lower entropies are selected and combined with the corresponding reciprocals of their entropies.

The proposed model is compared with several state-of-the-art visual attention models including SVO, Goferman's model, CBsal, AIM, SR, and ITTI's model. Experimental results show that the focus regions defined by Goferman's model, ITTI's model, SR, and AIM models are not always concentrated on the target and the backgrounds are not notably inhibited. The contrast between targets and backgrounds in the saliency maps obtained by SVO is relatively low. The CBsal model cannot highlight salient regions accurately when the target is tiny. The proposed method gives prominence to the valuable regions with high preciseness and restrains disturbances in the environment powerfully. The ROC, AUC, CC, and NSS scores of the seven visual attention models are calculated on four popular datasets and the experimental results quantitatively certificated the superiority of the proposed model.

Some theoretical arguments are given in the Supplementary discussion section, the section after Methods.

## Methods

### Connecting matrix learning

**Figure 6:**
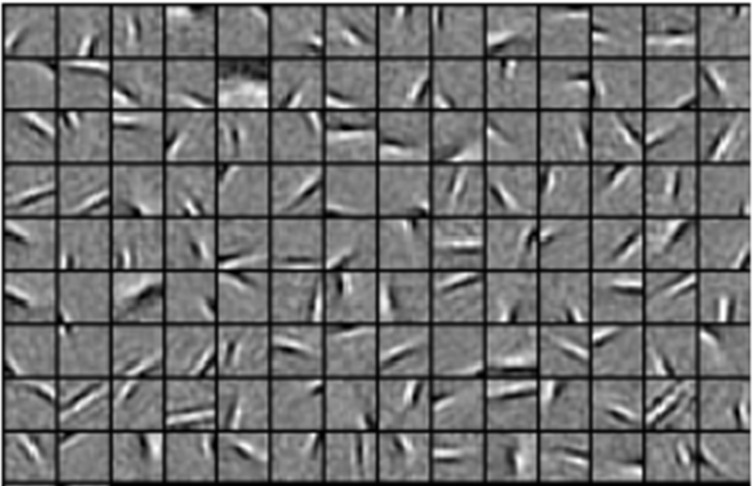
Well-trained connecting weight matrixes. We selected 126 connecting weight matrixes (i.e., RFs) randomly. Each square describes an oriented and localized Gabor-like RF of a specified V1 neuron. The gray pixels in each square represent zero, the lighter pixels correspond to positive values, and the darker ones indicate negative values. The localized, oriented, and band-pass RFs of neurons are somewhat like Gabor filters which is consist with that of accurate predictions of V1 RFs.

The connecting matrix of V1 neurons is trained before the computation of neuron responses. The E-I Net model^48^ is adopted in this process. 1000 patches randomly sampled from gray-scale natural images are whitened and normalized to learn Gabor-like RFs. The sparseness of neuron responses together with the error between the original and recovered images are the main factors to be considered as training rules. The size of patches is set as 14*14 (i.e., the dimension of input stimuli is 196) and 128 weight matrixes are learned finally. More details about the processing of the E-I Net model can be found in Ref. 48. An example of the learned RFs is presented in Figure 6. Neuron responses can be calculated using the learned RFs. The error between the original image and the image recovered with the corresponding neuron responses is minimized (see Result).

### Color space transformation

Different color spaces including RGB, CIELAB, HSI, and YIQ are utilized for color image processing. We have to transform the original images from RGB into other spaces. The conversions among them are given as follows.

1. *Conversion from RGB to CIELAB.* In the CIELAB color space L is the luminance channel, A is the red-green channel, and B is the yellow-blue channel. Firstly, the RGB space is converted to the XYZ space with the formula shown as follows.
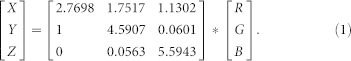
Then the XYZ space is converted to the CIELAB space with the following formula.
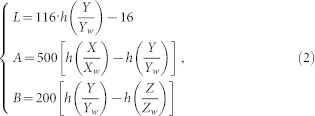
where *X_w_* = 95.04, *Y_w_* = 100.00, *Z_w_* = 108.89, and
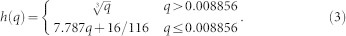
2. *Conversion from RGB to HSI.* In the HSI color space H is hue, which describes a pure color. S is saturation, which gives the degree to which a pure color is diluted by white light. I is intensity. The conversion can be achieved as follows.
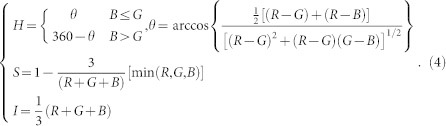
3. *Conversion from RGB to YIQ.* In the YIQ color space, Y represents luminance or intensity, while I and Q (in phase and quadrature) represent the chrominance or color information of an image. The relationship between RGB and YIQ is given as follows.
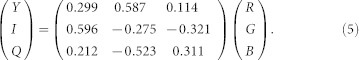

### Saliency detection and combination

Statistical histograms are built for each dimension of neuron responses. The number of bins in the histogram is set as 1000, which can be modified. With the histograms, likelihoods of the responses can be obtained. Afterwards, saliency is computed with self-information^40^ shown as:
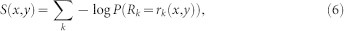
where *S* (*x, y*) is the saliency value of pixel (*x, y*), *r_k_* (*x, y*) is the *k* th dimension of neuron responses to pixel (*x, y*). Images in all separate channels of the four color spaces together with the gray-scale image are imported to the saliency detection part. The saliency sub-map with the lowest entropy is selected from each color space. The entropy of a saliency sub-map is defined as follows.
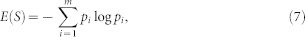
where *i* ∈[1, *m*] are all values in the saliency sub-map, *m* is the maximum value. *p_i_* is the probability of value *i* and it is computed with the histogram.

Afterwards, four selected saliency sub-maps together with the saliency sub-map obtained by the gray-scale image are linearly combined with their coefficients computed as:
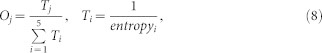
where *i* = 1, ...,5 representing the saliency maps extracted by four color channels and the gray-scale image. *entropy_i_* is the entropy of the *i* th saliency sub-map, and *O_j_* is the weight of the *j* th channel, *j* = 1, ..., 5.

## Theoretical arguments

Theoretical arguments of the proposed visual attention model are presented in three major aspects: *the color information utilization*, *the saliency measuring principle*, and *the explanation for entropy adoption*. The proposed model uses four different color spaces to ensure approximate perceptual uniformity or invariance to different conditions. We assume that all original images are stored in the RGB color space. Unfortunately, the RGB color space does not fully correspond to the space in which the human brain processes colors. In addition, illumination and colors are nested in the RGB space, which makes the processing in this space inconvenient. Therefore, other color spaces are considered in the proposed model. However, the best color space for saliency extraction is still uncertain. Distinct results can be obtained for different scenes in spite of using a certain color space. Thus, Three other color spaces together with RGB are used to extract saliency simultaneously.

There are many kinds of color spaces that can be classified into three categories according to the color perception^57^: the mixture-type color space, the luminance/chrominance-type color space, and the hue/saturation/luminance-type color space. Three color spaces in different categories are considered to make comprehensive use of color information. Thus the RGB color space in the first class, the CIELAB color space belonging to the second class, and the HSI color space in the last class are chosen. RGB is a color space in which original images are generally stored and it can be converted into other spaces conveniently. CIELAB is independent from the equipment and it is flexible to deal with the illumination changes. The intensity component and color information (hue and saturation) are decoupled in the HSI space. The YIQ color space takes advantage of human eye^58^ and it is more sensitive to intensity changes than to saturation or hue changes. Therefore, these four color spaces are chosen to compute saliency parallelly. Conversion relationships among RGB and the other three color spaces are given in the section of Methods in detail.

In free-viewing natural scenes, the saliency value of a pixel is related to the appearance and location of it. The prior knowledge of the obsever has influence on attention allocation^59^. The interesting probability of a pixel (*x, y*) can be computed with the Bayes theorem:
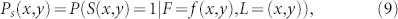
where *S* (*x, y*) = 1 indicates that the pixel (*x, y*) is salient. *F* is features of the pixel and *L* is the location of the pixel. As the features of a pixel are generally independent from the location of it, the computation of *P_s_* (*x, y*) can be rewritten as follows.
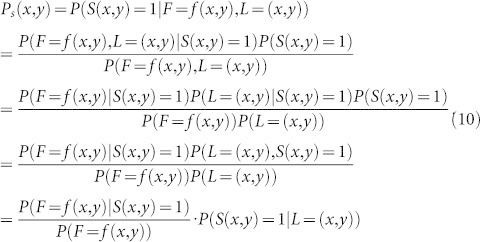
The log probability is estimated to compare this probability across different locations in an image. Therefore, log*P_s_* (*x, y*) is used to present the saliency value of the pixel (*x, y*), which is given as follows.
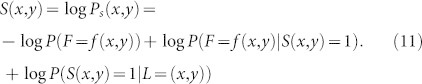
The first term in (11) represents the self-information of the random variable *F*. It is independent of any prior knowledge about the target class and depends only on the visual features observed at the point. The second term favors features that are consistent with the prior knowledge of the target features. The last term describes the prior location in which the target is likely to appear and it is independent of visual features. We suppose that all information is from current image and there is no prior guide information in bottom-up visual attention processing. Thus, the second and third terms in (11) are ignored in the proposed bottom-up visual attention model. The saliency map of an image is computed based on the neuron responses of it as the features of pixels are represented by neuron responses in the proposed model. Neuron responses in area V1 are approximately independent. The self-information of neuron responses at pixel (*x, y*) can be presented as:
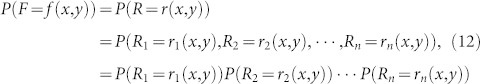
where *R* denotes neuron responses and *r_k_* (*x, y*), *k* = 1, ..., *n* represents the *k* th response at pixel (*x, y*). *n* is the dimension of neuron responses. The saliency value at pixel (*x, y*) can be computed as follows.
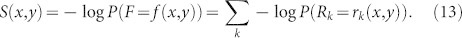
There should be a criterion to evaluate the quality of the saliency sub-maps computed in each channel of all color spaces, and combine them into the final saliency map. Entropy of a saliency sub-map is employed as an evaluation criterion. Entropies are also used to compute combining weights of the saliency sub-maps, which is distinct from the use of entropy in other visual attention models^60^. A saliency map can be considered as a probability map. Entropy is a measure of the uncertainty in the outcome of a random variable. Generally, regions of interest in an expected saliency map should be strengthened largely while the background should be suppressed. Values in the histogram of a desired saliency map would cluster around certain values. Under this circumstance, the entropy of a saliency map would be very low. Therefore, the saliency sub-map with the lowest entropy is selected from the three channels of each color space. Finally the saliency sub-map extracted from the gray-scale image and the saliency sub-maps chosen from four color spaces are combined. The combining weights are computed as the reciprocals of corresponding entropies. Additionally, the combining weights are normalized.

## Author Contributions

All authors have made contributions to this paper. H.D. and X.W. designed the whole work, produced all the data, and wrote the paper. H.D. supervised the whole work and contributed to the manuscript preparation. X.W. made simulations. All authors discussed the results and reviewed the manuscript.
